# Age and sex-dependent alteration in AMPA receptor density in living human brain

**DOI:** 10.1007/s00259-026-07963-7

**Published:** 2026-06-22

**Authors:** Mai Hatano, Waki Nakajima, Shohei Tsuchimoto, Tetsu Arisawa, Yuuki Takada, Tsuyoshi Eiro, Hiroki Abe, Sadamitsu Ichijo, Yu Fujimoto, Akane Sano, Shariful A. Syed, Hideaki Tani, Nobuhiro Nagai, Teruki Koizumi, Shinichiro Nakajima, Kie Nomoto-Takahashi, Yohei Ohtani, Masahiro Jinzaki, Yoji Hirano, Ryo Mitoma, Shunsuke Tamura, Shingo Baba, Osamu Togao, Hirotaka Kosaka, Hidehiko Okazawa, Yuichi Kimura, Masaru Mimura, Hiroyuki Uchida, Takuya Takahashi

**Affiliations:** 1 Department of Physiology, Yokohama City University Graduate School of Medicine, Yokohama, 236-0004 Japan; 2 Division of Neural Dynamics, Department of System Neuroscience, National Institute for Physiological Sciences, Okazaki, Aichi 444-8585 Japan; 3 Radioisotope Research Center, Yokohama City University Graduate School of Medicine, Yokohama, 236-0004 Japan; 4 Department of Psychiatry, Yokohama City University Graduate School of Medicine, Yokohama, 236-0004 Japan; 5 Department of Neuropsychiatry, Keio University School of Medicine, Tokyo, 160-8582 Japan; 6 Department of Radiology, Keio University School of Medicine, Tokyo, 160-8582 Japan; 7 Department of Psychiatry, Division of Clinical Neuroscience, Faculty of Medicine, University of Miyazaki, Miyazaki, 889-1692 Japan; 8 Department of Neuropsychiatry, Graduate School of Medical Sciences, Kyusyu University, Fukuoka, 812-8582 Japan; 9 Institute of Industrial Science, The University of Tokyo, Tokyo, 153-8505 Japan; 10 Department of Health Sciences, Graduate School of Medical Sciences, Kyusyu University, Fukuoka, 812-8582 Japan; 11 Department of Clinical Radiology, Graduate School of Medical Sciences, Kyusyu University, Fukuoka, 812-8582 Japan; 12 Department of Neuropsychiatry, School of Medical Sciences, University of Fukui, Fukui, 910-1193 Japan; 13 Biomedical Imaging Research Center, University of Fukui, Fukui, 910-1193 Japan; 14 Faculty of Informatics, Cyber Informatics Research Institute, Kindai University, Higashi, Osaka, 577-8502 Japan; 15 The International Research Center for Neurointelligence, Institutes for Advanced Study, University of Tokyo, Tokyo, 113-8654 Japan

**Keywords:** AMPA receptor, PET imaging, Synaptic aging

## Abstract

**Purpose:**

The excitatory glutamate α-amino-3-hydroxy-5-methyl-4-isoxazole propionic acid receptors (AMPARs) play a pivotal role in neurotransmission and neuronal function. However, the effects of age and sex on AMPAR distribution in the living human brain and their associations with cognitive function remain unclear. The purpose of this study was to characterize age- and sex-dependent changes in brain AMPAR density and their relationships with cognitive performance in healthy individuals.

**Methods:**

Using a positron emission tomography tracer for AMPAR, [^11^C]K-2, we imaged 143 healthy participants aged 20–79 years. AMPAR density was evaluated using standard uptake value ratios with white matter as a reference. Age- and sex-related changes in AMPAR density were assessed across the brain, hierarchical clustering was used to characterize sex-dependent regional patterns of age-related change, and associations with cognitive performance were examined using the Repeatable Battery for the Assessment of Neuropsychological Status (RBANS).

**Results:**

Age-dependent differences in cell-surface AMPAR density was observed across most brain regions. Females in their 50 s showed a surge in the upregulation of AMPAR density across brain. Hierarchical clustering revealed five distinct age-related trajectories, featuring marked sex-dependent regional patterns. AMPAR density was positively associated with cognitive performance; delayed memory correlated with whole-brain AMPAR density in both sexes, whereas other cognitive domains showed sex-specific regional associations.

**Conclusions:**

These findings demonstrated age- and sex-related alteration of AMPAR distribution and propose a model of AMPAR related synaptic aging in the living human brain over the life span. Furthermore, they may help to elucidate the pathophysiology of neurodegenerative disorders.

**Supplementary Information:**

The online version contains supplementary material available at https://doi.org/10.1007/s00259-026-07963-7.

## Introduction

Brain functions, such as cognitive function, decline with age, even without pathological changes. A large body of basic research has been conducted on the aging brain using experimental animals [1–4]. However, the profiling of synaptic information of the living human brain throughout life is required due to the differences in the brain size, complexity of information processing, and the life span. The synaptic information of healthy human brains at different ages helps to delineate the illness states of the brain.

The glutamatergic α-amino-3-hydroxy-5-methyl-4-isoxazole propionic acid receptor (AMPARs) is one of the most important molecules to mediate neurotransmission [5–7]. Given their critical role in synaptic function, understanding age-dependent molecular characteristics of AMPAR density on the cell surface may provide crucial information to elucidate age-related functional decline. To begin with, there are limitations in postmortem brains for assessing healthy aging, and it has been technically impossible to identify quantitative changes in AMPARs in living human brains. We recently developed a positron emission tomography (PET) tracer, [^11^C]K-2, that visualizes AMPARs in the living human brain [8]. We have also confirmed the safety and efficacy of [^11^C]K-2 by in vivo dosimetry [9] and presented that [^11^C]K-2 reflects AMPAR density on the cell surface which is functionally crucial fraction [10]. Recently, we characterized the distribution of AMPARs in patients with depression, schizophrenia, bipolar disorder, autism spectrum disorder and epilepsy [11, 12].

In this study, we aimed to determine age-dependent differences in AMPAR density in healthy participants using [^11^C]K-2. The results revealed that AMPAR decreases with age in most brain regions. Interestingly, AMPAR density exhibited transient increase in females in their 50 s. Using a hierarchical clustering method, the patterns of AMPAR reduction in the brain were divided into five groups, with males and females showing different characteristic patterns. Furthermore, AMPAR density decreased with cognitive decline. These findings provide a normative baseline for AMPAR changes across the lifespan, which may serve as a foundation for distinguishing between pathological and normal aging processes and potentially identify new therapeutic targets for age-related cognitive decline. It may also be important for future understanding of mental disorders as well as dementia (especially mild cognitive impairment) and age-related diseases.

## Materials and methods

### Inclusion and exclusion criteria of healthy participants

The first study (UMIN000020975) included healthy male participants aged 20–40 years who could provide informed consent and fulfill the following criteria: body mass index of 18.5–25.0, laboratory blood test results within normal limits, negative blood results for hepatitis B virus, hepatitis C virus, HIV, and syphilis. The exclusion criteria were a past or current history of any psychiatric disorder, current or past smoker who quit smoking within 6 months, presence of pathological finding(s) detected by MRI, having contraindications for MRI scan, or use of any drugs.

The second study (UMIN000025132) included healthy male participants aged 30–79 years who could provide informed consent and did not fulfil any diagnostic criteria for psychiatric conditions according to the diagnostic and statistical manual of mental disorders (DSM)-IV [13] using the SCID-I/DSM-IV [14], DSM-5 [15] or ICD-10 [16]. In addition, we excluded those with a history of epilepsy, positive urine drug screen for illicit drugs, contraindications for MRI scan, significant neurological or general medical conditions, or abnormal laboratory test values of serum creatinine ≥ 1.5 mg/dl, aspartate aminotransferase ≥ 150 IU/L, or alanine aminotransferase ≥ 150 IU/L, as well as those receiving treatment with perampanel.

The third study (jRCTs031200083) had similar inclusion criteria as the second study, except the age range (20–49 years) and sex (men and women were included). The exclusion criteria were the same as those in the second study, with the addition that pregnant women, nursing mothers, and women planning to become pregnant were excluded.

The fourth study (jRCTs031210312) included healthy participants aged 60–79 years who　could provide informed consent, did not have dementia, or meet the definition of epilepsy. The exclusion criteria were a history of craniotomy, contraindications for MRI, participating in another clinical trial or clinical research within 7 days prior to enrollment, taking perampanel within 28 days prior to enrollment, and inability to remain in bed during PET and MRI examinations.

The demographic characteristics of the participants are presented in Supplementary Table S1 and S2.

### PET and MRI imaging

The participants underwent a PET scan using [^11^C]K-2 and an MRI scan. [^11^C]K-2 was synthesized locally at each site in accordance with the ordinance of good manufacturing practices. The injected dose of [^11^C]K-2 was 376.5 ± 11.5 MBq. PET scans were performed on all PET scanners with a brain tumor (BT) phantom (Itoi Factory Inc.) to validate reconstruction settings, yielding PET images of uniform resolution across scanners [11]. Detailed scanner specifications, acquisition parameters, and reconstruction settings for each site are provided in the Supplementary Methods. All PET images were subsequently smoothed with a 5 mm Gaussian kernel during preprocessing to harmonize the effective spatial resolution across datasets. The number of participants for each scanner is summarized in Supplementary Table S2.

### Standard uptake value ratio (SUVR)

The SUVR images of [.^11^C]K-2 were obtained by dividing the radioactivity values of each brain region by the white matter radioactivity values measured 30 and 50 min after tracer injection (SUVR_30–50 min_). Since AMPAR is not expressed in the white matter, SUVR_30–50 min_ reflects the regional AMPAR density. A previous study showed that SUVR_30–50 min_ correlated with AMPAR density quantified by biochemical assay in surgically resected human brain tissue [8], indicating SUVR_30–50 min_ reflects regional AMPAR density. Additionally, the total distribution volume in white matter, where AMPAR is not expressed, was lowest among the various brain regions [8], supporting its use as a reference. The Volume of Interest (VOI) of white matter was obtained using Statistical Parametric Mapping (SPM) 8 by fulfilling the following voxel value conditions: the probability of white matter presence was > 0.9, the 8 mm-smoothed gray matter was < 0.05, and the 8 mm-smoothed cerebrospinal fluid was < 0.05 [8]

### Normalizing PET images for VOI analysis

VOI analyses were conducted on 143 healthy participants (87 men, mean age: 48.7 ± 17.2 years). For each participant, PET data were used to generate summed PET images (30–50 min post-injection of [^11^C]K-2). The 30–50 min summed PET images and T1-weighted MRI images were normalized to Montreal Neurological Institute (MNI) space using the PMOD PNEURO tool v3.7 (PMOD Technologies), enabling the use of the Hammersmith atlas. VOIs were automatically derived based on the most likely localization of brain areas encoded in the Hammersmith atlas [17].

### Regional SUVR analysis and age-related pattern clustering

Regional SUVR values were extracted from normalized PET images using the Hammersmith atlas. In the present study, eight regions corresponding to white matter and ventricular structures were excluded, resulting in 75 Gy matter regions for analysis. Participants were stratified into six age groups by decade (20 s through 70 s). Mean SUVR_30–50 min_ values were calculated for each brain region within each age group and centralized by subtracting each region’s overall mean across all age groups to highlight temporal patterns independent of regional baseline differences. Hierarchical cluster analysis was performed on these centralized patterns using Euclidean distance and Ward's linkage method. A distance threshold of 0.6 was applied based on visual inspection of the dendrogram, yielding five distinct clusters of regions with similar age-related SUVR trajectories.

### Normalizing PET images for correlation analysis between RBANS score and SUVR

Voxel-wise analysis using SPM12 was performed on 106 healthy participants who had obtained scores in RBANS [18] in four clinical studies (57 men, 41.3 ± 12.2 years). Participants underwent the RBANS test on the day of the PET scan. These assessments were performed by one of the trained investigators who was blinded to the PET and MRI data. The average RBANS scores of the healthy participants were: 98.2 ± 14.0 (total), 29.3 ± 4.2 (List learning), 18.5 ± 3.5 (Story memory), 17.5 ± 2.6 (Figure copy), 18.1 ± 2.2 (Line orientation), 6.8 ± 2.2 (List recall), 19.4 ± 0.9 (List recognition), 10.2 ± 2.1 (Story recall) and 13.7 ± 4.2 (Figure recall) (Supplementary Fig. S1 and Supplementary Table S1). RBANS index scores are composite measures derived from the 12 subtests, comprising immediate memory, visuospatial constructional ability, attention, language, and delayed memory, and are adjusted for age. Because this study aimed to examine the association between age-related differences in cognitive function and SUVR, we avoided the influence of age-adjusted normative scoring in RBANS index scores. Therefore, five index scores were calculated using raw subtest scores. Specifically, Z-scores were computed for each subtest across participants, and index scores were calculated as the mean of the corresponding subtest Z-scores within each index.

For each participant, a summed PET image was acquired 30–50 min after the injection of [^11^C]K-2. SUVR_30–50 min_ images were normalized to the mean white matter value. A normalization template was generated from the 3D T1WI of all participants using the high-dimensional nonlinear warping algorithm, DARTEL [19], to achieve more accurate inter-subject anatomical alignment. Using the template in SPM 12, the SUVR_30–50 min_ images were spatially normalized to the MNI standard space while preserving the concentration to avoid volume effects. The spatially normalized images were further smoothed with an 8 mm Gaussian kernel.

### Statistical analysis

For the analysis of age-related changes in AMPAR density across the whole brain, a one-way analysis of variance (ANOVA) with post-hoc Tukey's test was used. For the analysis of sex differences in changes in AMPAR density, a two-way ANOVA followed by post-hoc Sidak’s test was used. *P* < 0.05 was statistically significant in both analyses. We did not correct multiple comparisons between brain regions, as the regional analyses were conducted in an exploratory manner to characterize whole-brain spatial patterns of age- and sex-related differences in AMPAR distribution, rather than to test predefined region-specific hypotheses.

The association between SUVR_30–50 min_ and RBANS scores was evaluated using a multiple regression model implemented in SPM12. Site was included as a covariate in all voxel-wise analyses. For the analysis based on the five RBANS index scores, to account for multiple comparisons across the five indices, statistical significance was defined using a conservative voxel-level threshold of *P* < 0.001 (uncorrected), with a false discovery rate correction applied at *P* < 0.05 (cluster-level inference, false discovery rate correction) to account for multiple comparisons across all in-mask voxels. For the analysis based on the 12 RBANS subtest scores, statistical significance was defined as* P* < 0.025 at the peak level (uncorrected), with a false discovery rate correction applied at *P* < 0.05 (cluster-level inference, false discovery rate correction) to account for multiple comparisons across all in-mask voxels. Given the exploratory nature of this analysis, no correction for multiple comparisons across the 12 RBANS subtests was applied.

## Result

### Age dependent differences in AMPAR density

**Fig. 1:**
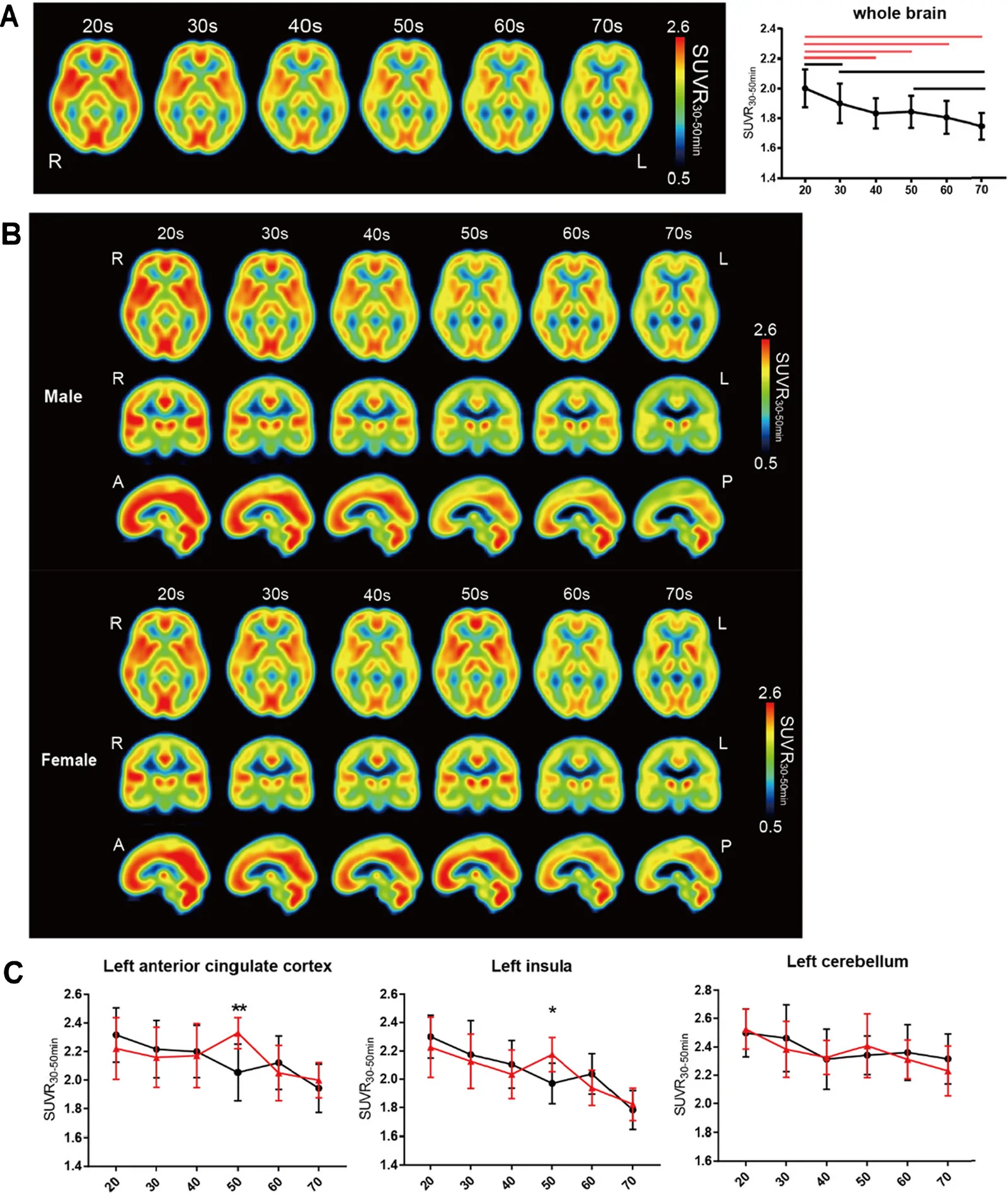
**Age-related decline in AMPAR density. (A)** Average SUVR_30–50 min_ images of healthy participants in their 20 s, 30 s, 40 s, 50 s, 60 s, and 70 s in axial slices (left). Changes in SUVR_30–50 min_ from the 20 s to 70 s in the whole brain (right). Statistical significance was calculated using one-way analysis of variance with Tukey’s multiple comparisons test. Black and red lines indicate P < 0.05 and P < 0.001, respectively. **(B)** Average SUVR_30–50 min_ images for males and females in their 20 s, 30 s, 40 s, 50 s, and 60 s in axial, coronal, and sagittal slices. **(C)** Changes in SUVR_30–50 min_ from the 20 s to 70 s by sex in the left ACC, left insula, and left cerebellum. The red and black lines indicate females and males, respectively. Data are expressed as mean ± SD. Statistical significance was calculated using two-way analysis of variance with Sidak’s multiple comparison test. Uncorrected for multiple comparisons across regions. *P < 0.05, **P < 0.01. ACC; anterior cingulate cortex

To examine age-dependent differences in AMPAR density, we performed PET imaging with [^11^C]K-2 in healthy participants aged 20–79 years. Then, we obtained standard uptake value ratio using white matter as a reference during 30–50 min after injection of [^11^C]K-2 (SUVR_30–50 min_) images. Previous studies have shown that SUVR_30–50 min_ is closely equivalent to non-displaceable binding potential (*BP*_ND_), an index of receptor density, and that SUVR_30–50 min_ is strongly correlated with AMPAR density measured in surgically resected brain tissue. Based on these finding, SUVR_30–50 min_ reflects the AMPA receptor density [8, 10]. The SUVR_30–50 min_ images were averaged in each age group. We found gradual age-dependent differences in whole-brain SUVR_30–50 min_ (Fig. 1A and Supplementary Fig. S2).

We compared SUVR_30–50 min_ of the images of males and females across different age groups to examine whether there was a sex-dependent difference in AMPAR density (Fig. 1B). In males, the SUVR_30–50 min_ across the entire brain decreased with age, whereas a transient increase was observed in females in their 50 s (Fig. 1B). Line graphs depicting age-related changes in SUVR_30–50 min_ for each brain region by sex revealed that in several regions, females demonstrated a transient increase of SUVR_30–50 min_ in their 50 s compared with males (Fig. 1C and Supplementary Figs. S3-6), particularly in the left straight gyrus, posterior orbital gyrus, left lateral orbital gyrus, left anterior superior temporal gyrus, left anterior medial temporal lobe, anterior cingulate cortex (ACC), left posterior cingulate gyrus (PCC), left insula, right subgenual ACC, substantia nigra and right nucleus accumbens. Conversely, no clear sex-related differences were observed in the parietal lobe, occipital lobe or cerebellum across all age groups (Fig. 1C and Supplementary Fig. S5).

### Sex-dependent differences in AMPAR density across all age groups with hierarchical clustering analysis

**Fig. 2:**
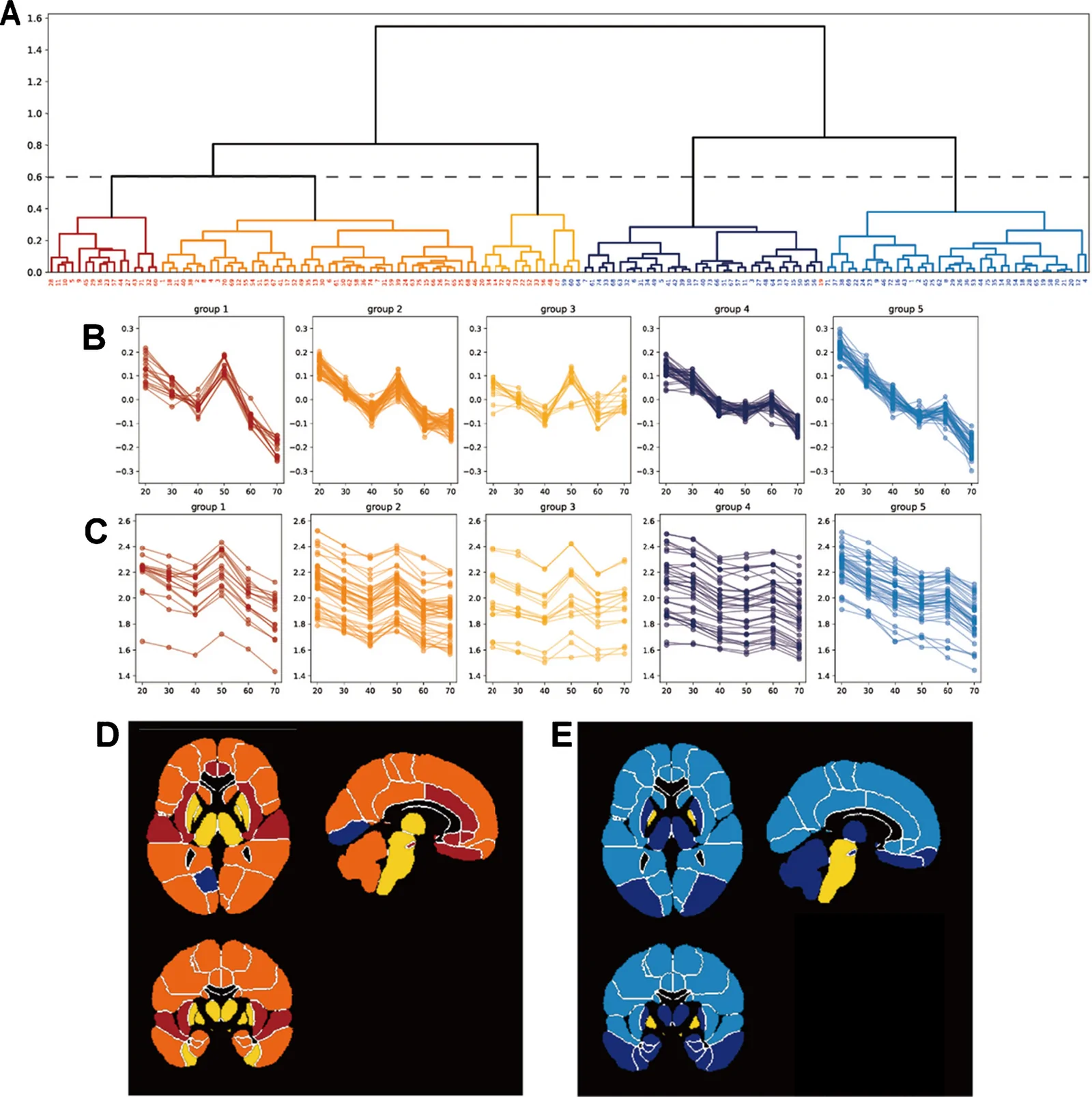
**Sex differences in AMPAR density.** **(A)** Dendrogram of the age-dependent pattern of change in SUVR_30–50 min_ in each brain region in males and females when the distance threshold was set to 0.6 using hierarchical cluster analysis. Red numbers indicate brain regions in females, while blue numbers indicate those in males. **(B)** Age-related changes in normalized SUVR_30–50 min_ for each group in the results of the hierarchical clustering analysis with a distance threshold of 0.6. **(C)** Age-related changes in SUVR_30–50 min_ for each group in the results of hierarchical clustering analysis with a distance threshold of 0.6. **(D)** Brain map of the female showing regions color-coded for each group in the results of hierarchical clustering analysis with distance threshold 0.6. Red: Group 1, orange: Group 2, yellow: Group 3, dark blue: Group 4. **(E)** Brain map of the male showing regions color-coded for each group in the results of hierarchical clustering analysis with distance threshold 0.6. Yellow: Group 3, dark blue: Group 4, sky blue: Group 5

To further investigate whether the patterns of age-related changes in AMPAR density differed between both sexes, we performed hierarchical clustering analysis on SUVR_30–50 min_ values from 150 anatomical regions (75 regions per sex) across all age groups (Fig. 2A and Supplementary Fig. S7A). To compare the trajectories of age-related changes independent of regional density levels, SUVR_30–50 min_ for each brain region were normalized by the mean value across all age groups (20 s-70 s) before clustering. This procedure allowed us to highlight how each brain region changes relative to its own baseline, making the patterns of age-related directly comparable across cortical and subcortical structures. Hierarchical agglomerative clustering revealed five distinct clusters of brain regions when the distance threshold was set at 0.6. Group 1 consisted solely of brain regions in females that showed a decrease in SUVR_30–50 min_ from 20 to 40 s, followed by a transient increase in their 50 s and a sharp decline from their 60 s–70 s (Fig. 2 A-C and Table 1). Group 2 also included only brain regions in females, showing an initial decline in SUVR_30–50 min_ from the 20 s to 40 s, a transient increase in the 50 s, and relatively stable levels in the 60 s–70 s (Fig. 2 A-C and Table 1). Group 3 comprised brain regions in both males and females that exhibited minimal changes in SUVR_30–50 min_ across all age groups (Fig. 2 A-C and Table 1). Group 4 was composed predominantly of brain regions in males, showing a gradual decline from the 20 s to 70 s, except for a single region in females, the right lingual gyrus (Fig. 2 A-C and Table 1). Group 5 included only brain regions in males that exhibited a rapid decline in SUVR_30–50 min_ from the 20 s to 70 s (Fig. 2 A-C and Table 1). Overall, the hierarchical clustering results confirmed sex-dependent differences in the patterns of age-related SUVR_30–50 min_ decline across the brain regions. Furthermore, in males, the left and right brain regions belonged to the same cluster, indicating no lateral differences in the patterns of SUVR_30–50 min_ decline (Table 1).

**Table 1:** The brain regions belonging to each group in the results of hierarchical clustering analysis with a distance threshold of 0.6

Group	Brain regions
Group 1	*Insula (L, R), ACC (L, R), Pregenual ACC (L, R), Subgenual ACC (L, R), Straight gyrus (L, R), Nucleus accumbens (L, R),* *Posterior superior temporal gyrus (L, R), Substantia nigra (R),* *Posterior orbital gyrus (R)*
Group 2	*Superior frontal gyrus (L, R), Middle frontal gyrus (L, R), Inferior frontal gyrus (L, R), Medial orbital gyrus (L, R), Lateral orbital gyrus (L, R), Posterior orbital gyrus (L) Precentral gyrus (L, R), PCC (L, R), Superior parietal gyrus (L, R), Inferiolateral reminder of parietal lobe (L, R), Postcentral gyrus (L, R),* *Cuneus (L, R), Lingual gyrus (L), Inferiolateral reminder of occipital lobe (L, R), Anterior medial temporal lobe (L, R), Anterior superior temporal gyrus (L, R), Posterior temporal lobe (L, R), Middle and inferior temporal lobe (L, R),* *Inferior temporal lobe (L, R), Hippocampus (L, R), Parahippocampal gyrus (L, R), Amygdala (L, R), Caudate (L, R), Cerebellum (L, R)*
Group 3	*Putamen (L, R), Thalamus (L, R), Anterior orbital gyrus (L, R), Fusiform gyrus (L, R), Substantia nigra (L), Globus pallidus (L, R), Brain stem,* Globus pallidus (L, R), Brain stem
Group 4	Straight gyrus (L, R), Anterior orbital gyrus (L, R), Medial orbital gyrus (L, R), Lateral orbital gyrus (L, R), Inferiolateral reminder of occipital lobe (L, R), Anterior medial temporal lobe (L, R), Middle and inferior temporal lobe (L, R), Inferior temporal lobe (L, R), Hippocampus (L, R), Parahippocampal gyrus (L, R), Fusiform gyrus (L, R), Amygdala (L, R), Putamen (L, R), Thalamus (L, R), Nucleus accumbens (L, R), Substantia nigra (L, R), Cerebellum (L, R), *Lingual gyrus (R)*
Group 5	Insula (L, R), ACC (L, R), Pregenual ACC (L, R), Subgenual ACC (L, R), PCC (L, R), Superior frontal gyrus (L, R), Middle frontal gyrus (L, R), Inferior frontal gyrus (L, R), Posterior orbital gyrus (L, R), Precentral gyrus (L, R), Postcentral gyrus (L, R), Superior parietal gyrus (L, R), Inferiolateral reminder of parietal lobe (L, R), Cuneus (L, R), Lingual gyrus (L, R), Anterior superior temporal gyrus (L, R), Posterior superior temporal gyrus (L, R), Posterior temporal lobe (L, R), Caudate (L, R)

Italic letters indicate brain regions in females

Next, we generated sex-specific brain maps that were color-coded by clusters (Fig. 2D and E and Supplementary Fig. S7B and C). In males, the frontal, parietal, and occipital regions, along with the ACC, insula, PCC, and caudate, showed a rapid decline in SUVR_30–50 min_, while the temporal lobe, cerebellum, and basal ganglia exhibited a gradual decline. In addition, the globus pallidus and brainstem showed minimal change with age (Fig. 2E and Supplementary Fig. S7C). In females, the ACC, insula, and nucleus accumbens were assigned to the rapidly declining cluster between ages 60 and 70. In contrast, most neocortical regions remained stable during this period. The putamen, thalamus, globus pallidus, and brainstem showed minimal changes across age groups (Fig. 2D and Supplementary Fig. S7B). Therefore, the rapid decline in AMPAR was widespread across brain regions in males but limited to specific regions in females. Notably, the globus pallidus and brainstem remained stable in both sexes.

### Relationship between cognitive function and AMPAR density

To examine whether the relationship between cognitive function and AMPAR density differs by sex in healthy participants, we analyzed the correlations between SUVR_30–50 min_ and each of the five index scores of Repeatable Battery for the Assessment of Neuropsychological Status (RBANS)—a test to assess cognitive function—separately for males and females. Five index scores were calculated based on standardized raw subtest scores, as described in the Methods. In addition, correlations with the raw scores of the 12 RBANS subtests were also examined and are presented in Supplementary Fig. S8. Age-related changes in each RBANS score by sex are shown as line graphs in Supplementary Fig. S1. No significant sex difference was observed in the decline in RBANS scores with age.

**Fig. 3:**
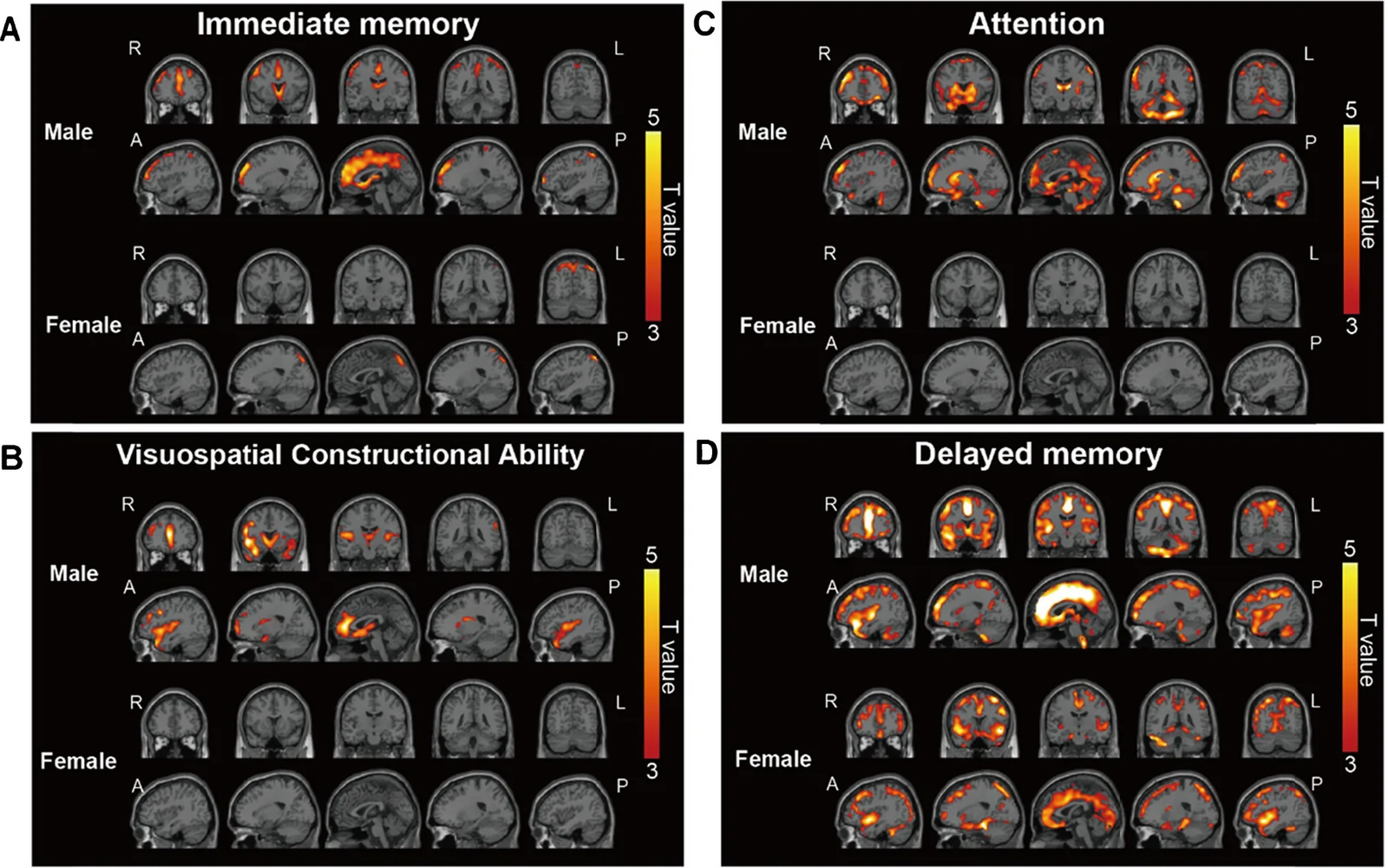
**Relationship between cognitive function and AMPAR density in healthy participants. (A)-(I)** Brain region by sex with significant positive correlation between SUVR_30–50 min_ and each RBANS index score (*P* < 0.001, T > 3.0, one-tailed, FDRc, adjusted for site as a covariate). Significant clusters displayed on a sagittal slice. Visuospatial Constructional Ability and Attention scores in females were not significantly correlated with SUVR_30–50 min_. FDRc; false discovery rate correction

The immediate memory index showed a significant positive correlation with SUVR_30–50 min_ in the frontal lobe, ACC, PCC, precentral gyrus, postcentral gyrus, precuneus, caudate and thalamus in males. In females, significant positive correlations with SUVR_30–50 min_ were observed in the precuneus, superior parietal gyrus, superior occipital gyrus (Fig. 3A).

The visuospatial constructional ability index showed significant positive correlations with SUVR_30–50 min_ in the superior frontal lobe, ACC, superior temporal gyrus, insula, caudate and thalamus in males, whereas no significant correlations were found in females (Fig. 3B).

The attention index showed significant positive correlations with SUVR_30–50 min_ across the brain in males, particularly in the frontal lobe, ACC, PCC, precentral gyrus, postcentral gyrus, parietal lobe, occipital lobe, cerebellum, parahippocampal gyrus, fusiform gyrus, and basal ganglia (caudate, putamen, and pallidum), as well as the thalamus, whereas no significant correlations were found in females (Fig. 3C).

The language index showed no significant correlations with SUVR_30–50 min_ in both sexes.

The delayed memory index showed significant positive correlations with SUVR_30–50 min_ across the whole brain in males and females (Fig. 3D). While there was substantial overlap between sexes, male-specific correlations were observed in the caudate, whereas female-specific correlations were observed in occipital regions around the lingual gyrus.

Taken together, these findings demonstrated that several cognitive indices exhibited sex-dependent patterns of association with AMPAR density. In males, all indices except language showed significant positive correlations with AMPAR density, with widespread spatial patterns across the brain. In contrast, in females, significant associations were primarily observed in the immediate and delayed memory indices, with the immediate memory index showing a more regionally restricted and distinct pattern compared to males. The delayed memory index consistently showed widespread correlations with AMPAR density across the whole brain in both males and females, with partial overlap as well as sex-specific regional patterns.

## Discussion

In this study, we used [^11^C] K-2 PET imaging data of 143 healthy participants aged 20–79 years to provide the first comprehensive mapping of age-related changes in AMPAR density across the living human brain. Our findings demonstrate a widespread decrease in AMPAR density with aging in most brain regions, and the regional pattern of decline differs significantly between both sexes (Figs. 1 and 2). Previous studies in aged mice have reported reduced AMPAR density [20–24], however, the results have been inconsistent regarding regional specificity, and animal models have incompletely recapitulated human aging processes. Studies on post-mortem human tissues have compared AMPAR expression between diseased and healthy human brains [25–28]. However, age-related changes in AMPAR during normal aging have remained largely unexplored [29]. Our study addresses this critical knowledge gap by directly visualizing and quantifying region-specific age-dependent differences in AMPAR density in the living human brain.

Previous studies have not explored sex-related differences in changes in AMPAR density in the brain across individuals aged 20–79 years. This study is the first to demonstrate sex-related differences in AMPAR density in the brains of healthy participants. Notably, this study observed an increase in AMPAR density in females in their 50 s (Fig. 1B and C). Females typically undergo menopause around their 50 s, and menopause causes a decrease in estrogen production. Learning-dependent increases in hippocampal Ca^2^⁺-permeable AMPARs have been observed in ovariectomized female rats [30]. These findings raise the possibility that the increase in AMPAR density observed in females in their 50 s may be related to changes in estrogen levels and menopausal or perimenopausal status. In this study, we did not assess menopausal status, hormone levels or menstrual cycles; however, our findings provide a strong rationale for conducting further [^11^C] K-2 PET imaging studies in pre- and post-menopausal females, as well as patients with menopausal disorders and premenstrual syndrome.

Hierarchical agglomerative clustering has identified five distinct patterns in the age-related decline in AMPAR density (Fig. 2). In males, brain regions exhibiting rapid decline primarily comprise the cerebral cortex, while the subcortical structures predominantly show a gradual decline or no significant changes (Fig. 2E and Supplementary Fig. S7C). The cerebral cortex, which undergoes the most pronounced AMPAR reduction, is responsible for higher-order functions, including cognition, emotional processing, and language [31, 32]. In contrast, regions with more preserved AMPAR density, such as the hippocampus, amygdala, basal ganglia and cerebellum, regulate motor functions, emotional behavior, and other instinctive processes [33–35]. The brainstem, which shows minimal age-related changes, controls vital functions, such as respiration and body temperature [36, 37]. This regional specificity suggests that age-related AMPAR decline may be selectively attenuated in brain areas governing survival-critical functions, potentially representing an evolutionary preservation mechanism. In contrast, although AMPA receptor density tended to increase across brain regions in females in their 50 s, notable regional differences were observed. While most of the cerebral cortex maintained stable AMPA receptor density in individuals in their 60 s and 70 s, Group 1 regions—including the ACC and insula—exhibited a marked decline during this period (Fig. 2D and Supplementary Fig. S7B). Our previous studies have suggested that the reduction in AMPAR density in the ACC and insula may serve as a neurobiological substrate for psychiatric disorders such as bipolar disorder, schizophrenia, and autism spectrum disorder [11]. Considering the present findings, we hypothesize that the rapid decline in AMPAR density in the ACC and insula beyond age 50–59 contributes to vulnerability, which may increase the risk of psychiatric disorders, particularly dementia. Notably, previous reports have indicated that females are at a higher risk of developing dementia, particularly Alzheimer’s disease, than males [38, 39], suggesting a potential relationship between sex-specific AMPAR changes and disease susceptibility. Future longitudinal studies and fundamental research using animal models are necessary to validate this hypothesis.

In this study, we identified sex-specific patterns in the relationship between AMPAR density and cognitive function using RBANS indices and subtests. In females, the immediate memory index showed positive correlations with AMPAR density, primarily in specific regions, including the parietal lobe and occipital lobe (Fig. 3A and Fig. S8A,B). In contrast, delayed memory scores were positively correlated with AMPAR density across the whole brain (Fig. 3D and Fig. S8F-I). This pattern differed from that observed in males, in which immediate and delayed memory indices showed significant positive correlations with AMPAR density across the brain (Fig. 3A, D and Fig. S8A, B and F-I). These findings suggest that in females, different brain regions may be engaged depending on the stage of memory processing. Immediate memory may rely more on visual and sensory-related regions, whereas delayed memory may require a widespread neural involvement. In contrast, males may consistently use a broader network of brain regions across both memory stages. This may reflect sex-specific neural strategies in memory encoding and retrieval.

A key limitation of this study is that it employed a cross-sectional study design rather than a longitudinal design. We observed age-related changes in AMPAR density; however, these findings did not directly capture within-subject changes over time. Longitudinal studies on the same demographic across different ages would be necessary to confirm the trajectory of AMPAR alterations. Another limitation of this study is that information on menstrual cycle, menopausal status, and hormone levels was not collected in female participants. Therefore, the factors underlying the observed changes in females in their 50 s remain speculative, and further studies are required to clarify the underlying mechanisms.

## Conclusions

This study provides an important dataset that can serve as a reference for future research on AMPAR distribution in the human brain. Our findings provide a better understanding of how AMPAR changes with aging and cognitive decline, in healthy individuals and those with diseases. Since neurodegenerative disorders are a major challenge for aging populations, understanding these AMPAR changes could be key to developing better prevention and treatment strategies.

## Supplementary Information

Below is the link to the electronic supplementary material.Supplementary file1 (DOCX 4250 KB)

## Data Availability

The MRI and PET datasets analyzed during the current study are not publicly available due to ethical restrictions and the sensitive nature of human imaging data. However, the data are available from the corresponding author upon reasonable request and with appropriate institutional and ethical approvals.
