# Supplementary material for: Age and sex-dependent alteration in AMPA receptor density in living human brain
Source: Eur J Nucl Med Mol Imaging. 2026 Jun 22;53(11):6299–310. doi: 10.1007/s00259-026-07963-7 (PMC13526145; doi:10.1007/s00259-026-07963-7)
Supplement: Supplementary file 1 [file 259_2026_7963_MOESM1_ESM.docx]

**Supplementary Information Text**

**Supplementary Methods**

***PET and MRI imaging.***

***Yokohama City University Hospital:*** PET imaging was conducted using an Aquiduo scanner (TOSHIBA Medical) and a Celesteion PCA-9000A/2A (Canon Medical). The Aquiduo offered an axial field of view (FOV) of 240 mm with 80 contiguous 2.0 mm-thick slices. A 4.7-s transmission scan was used for attenuation correction (AC), followed by a 60-s intravenous injection of [^11^C]K-2. A 60-min emission scan was performed, divided into frames of 18 × 10 s, 2 × 30 s, 7 × 60 s, 1 × 2 min, 1 × 3 min, 3 × 5 min, and 3 × 10 min. Dynamic images were reconstructed using two-dimensional-ordered subsets expectation maximization (2D-OSEM) with four iterations, 14 subsets, a 128 matrix, a zoom of 2.8, and a 5.0 mm Gaussian filter. The Celesteion provided an axial FOV of 240 mm with 96 contiguous 2.0 mm-thick slices. A 15.2-s transmission scan was performed for AC, followed by a 60-s intravenous injection of [^11^C]K-2 and a 60-min emission scan consisting of 35 frames. Dynamic images were reconstructed using three-dimensional (3D)-OSEM + Time of Flight (TOF) with two iterations, 20 subsets, a 128 matrix, a zoom of 1.0, and a 5.0 mm Gaussian filter. An MRI scan was conducted for each participant using a GE DISCOVERY MR750 (General Electric Medical Systems) to ensure precise delineation of brain regions for data analysis. High-resolution 3D-T1-weighted images (T1WI) were obtained with the following parameters: voxel size = 0.9 × 0.9 × 0.9 mm, Repetition Time (TR)/Echo Time (TE) = 7.0/3.1 ms, flip angle (FA) = 8°, FOV = 220 mm, and matrix size = 256 × 256.

***Keio University Hospital:*** PET imaging was performed using a Biograph mCT Flow (Siemens Medical Solutions) with an axial FOV of 300 mm and 111 contiguous 2.0 mm-thick slices. A 6.56-s transmission scan was conducted for AC, followed by a 60-s intravenous injection of [^11^C]K-2 and a 60-min emission scan comprising 35 frames. Dynamic images were reconstructed using 3D-OSEM + TOF with four iterations, 24 subsets, a 200 matrix, a zoom of 2.0, and a 2.0 mm Gaussian filter. To support accurate brain region delineation for data analysis, participants also underwent an MRI scan on a MAGNETOM Prisma (SIEMENS Healthineers) at the University of Tokyo. High-resolution 3D-T1WI were acquired using a 3D MPRAGE protocol with a voxel size of 0.8 × 0.8 × 0.8 mm, TR/TE = 2400/2.22 ms, FA = 8°, FOV = 208 mm, and matrix size = 300 × 320.

***Kyushu University Hospital:*** PET imaging was conducted using a Biograph mCT Flow and a Biograph Vision (Siemens Medical Solutions). The Biograph mCT provided an axial FOV of 250 mm and 165 contiguous 1.0 mm-thick slices. A 9.38-s transmission scan was used for AC, followed by a 60-s intravenous injection of [11C]K-2 and a 60-min emission scan with 35 frames. Dynamic images were reconstructed using 3D-OSEM + TOF with five iterations, 21 subsets, and a 5.0 mm Gaussian filter. The Biograph Vision offered an axial FOV of 357 mm and 263 contiguous 1.0 mm-thick slices. A 26.67-s transmission scan was used for AC, followed by a 60-s intravenous injection of [^11^C]K-2 and a 60-min emission scan with 35 frames. Dynamic images were reconstructed using 3D-OSEM + TOF with eight iterations, five subsets, and a 5.0 mm Gaussian filter. MRI scans were performed on an Ingenia 3.0-T scanner (Phillips). High-resolution 3D-T1WI were acquired using a 3D MPRAGE protocol with a voxel size of 1.2 × 1.0 × 1.0 mm, TR/TE = 6.8/3.1 ms, FA = 9°, FOV = 170 mm, and matrix size = 256 × 256.

***University of Fukui Hospital:*** PET and MRI scans were performed simultaneously using a Signa PET/MRI (GE Healthcare). The Signa PET/MRI provided an axial FOV of 256 mm and 89 contiguous 2.78 mm-thick slices. For AC, a 3D radial MR acquisition with a zero-echo time (ZTE) method in the axial direction was performed with a FOV of 264 mm, matrix size of 110 × 110 × 116, voxel size of 2.4 × 2.4 × 2.4 mm, FA = 0.8°, number of excitations = 4, bandwidth = ± 62.5 kHz, and acquisition time of 41 s. The ZTE-AC method was previously described. A 60-s intravenous injection of [^11^C] K-2 was performed, followed by a 60-min emission scan comprising 35 frames. Dynamic images were reconstructed using 3D-OSEM + TOF with three iterations, 28 subsets, a 128 matrix, and a 3.0 mm Gaussian filter. Simultaneous high-resolution 3D-T1WI and other MR images were acquired with a voxel size of 0.9 × 0.9 × 0.9 mm, TR/TE = 8.5/3.3 ms, FA = 8°, FOV = 196 mm, and matrix size = 256 × 256.

For participants who underwent scanning at Keio University Hospital and the University of Fukui Hospital, dynamic PET images were processed using a 5 mm FWHM Gaussian filter to match the resolution of the PET images.

**Fig. S1. Age- and Sex-related differences in RBANS scores from the 20s to the 70s.**

Red line indicated females and black line indicated males. Data is expressed as mean ± SD. Statistical significance was calculated using two-way ANOVA with Sidak’s multiple comparison test.

**Fig. S2.** **Age-related decline in AMPAR density**

Average SUVR_30-50min_ images in healthy subjects in their 20s, 30s, 40s, 50s, 60s and 70s. Top shows axial slice, middle shows coronal slice and bottom shows sagittal slice.

**Fig. S3. Age- and Sex-related differences in SUVR_30-50min_ from the 20s to the 70s in frontal lobe.**

Changes in SUVR_30-50min_ from 20s to 70s by sex in the frontal lobe of the left and right sides respectively. Red line indicated females and black line indicated males. Data is expressed as mean ± SD. Statistical significance was calculated using two-way ANOVA with Sidak’s multiple comparison test. **P* < 0.05, ***P* < 0.01. Uncorrected for multiple comparisons across regions.

**Fig. S4. Age- and Sex-related differences in SUVR_30-50min_ from the 20s to the 70s in temporal lobe**

Changes in SUVR_30-50min_ from 20s to 70s by sex in the temporal lobe of the left and right sides respectively. Red line indicated females and black line indicated males. Data is expressed as mean ± SD. Statistical significance was calculated using two-way ANOVA with Sidak’s multiple comparison test. **P* < 0.05, ***P* < 0.01. Uncorrected for multiple comparisons across regions.

**Fig. S5. Age- and Sex-related differences in SUVR_30-50min_ from the 20s to the 70s in parietal lobe, occipital lobe, cerebellum and brain stem.**

Changes in SUVR_30-50min_ from 20s to 70s by sex in the parietal lobe, occipital lobe, cerebellum and brain stem. For brain regions other than the brainstem, graphs were presented separately for left and right. Red line indicated females and black line indicated males. Data is expressed as mean ± SD. Statistical significance was calculated using two-way ANOVA with Sidak’s multiple comparison test. **P* < 0.05, ***P* < 0.01. Uncorrected for multiple comparisons across regions.

**Fig. S6. Age- and Sex-related differences in SUVR_30-50min_ from the 20s to the 70s in cingulate cortex, insula and basal ganglia.**

Changes in SUVR_30-50min_ from 20s to 70s by sex in the cingulate cortex, insula and basal ganglia of the left and right sides respectively. Red line indicated females and black line indicated males. Data is expressed as mean ± SD. Statistical significance was calculated using two-way ANOVA with Sidak’s multiple comparison test. **P* < 0.05, ***P* < 0.01. Uncorrected for multiple comparisons across regions.

**Fig. S7. The result of hierarchical clustering analysis**

**(A)** Heat map and hierarchical clustering analysis by Euclidean distance generated from age-related SUVR_30-50min_ change patterns in each brain region. **(B)** Brain map of female showing brain regions color-coded for each group in the results of hierarchical clustering analysis with distance threshold 0.6 in axial, coronal, sagittal slices. Red: group 1, orange: group 2, yellow: group 3, dark blue: group 4. **(C)** Brain map of male showing brain regions color-coded for each group in the results of hierarchical clustering analysis with distance threshold 0.6 in axial, coronal, sagittal slices. Yellow: group 3, dark blue: group 4, sky blue: group 5.

**Fig. S8. Relationship between cognitive function and AMPAR density in healthy participants based on the raw scores of the 12 RBANS subtests**

(A)-(I) Brain region by sex with significant positive correlation between SUVR_30-50min_ and each subtest raw score of RBANS (*P* < 0.025, *T* > 2.0, one-tailed, FDRc, adjusted for site as a covariate). Significant clusters displayed on a sagittal slice. Line orientation scores in females were not significantly correlated with SUVR_30-50min_. FDRc; false discovery rate correction.

**Table S1. Demographic data of healthy participants.**

|  |  |  | **RBANS score** | | | | | | | | | | | |
| --- | --- | --- | --- | --- | --- | --- | --- | --- | --- | --- | --- | --- | --- | --- |
| **Participant** | **age** | **sex** | **List Learning** | **Story Memory** | **Figure Copy** | **Line Orientation** | **Picture Naming** | **Semantic Fluency** | **Digit Span** | **Coding** | **List Recall** | **List Recognition** | **Story Recall** | **Figure Recall** |
| **1** | **20** | **M** | **N/A** | **N/A** | **N/A** | **N/A** | **N/A** | **N/A** | **N/A** | **N/A** | **N/A** | **N/A** | **N/A** | **N/A** |
| **2** | **20** | **M** | **N/A** | **N/A** | **N/A** | **N/A** | **N/A** | **N/A** | **N/A** | **N/A** | **N/A** | **N/A** | **N/A** | **N/A** |
| **3** | **22** | **M** | **N/A** | **N/A** | **N/A** | **N/A** | **N/A** | **N/A** | **N/A** | **N/A** | **N/A** | **N/A** | **N/A** | **N/A** |
| **4** | **21** | **M** | **N/A** | **N/A** | **N/A** | **N/A** | **N/A** | **N/A** | **N/A** | **N/A** | **N/A** | **N/A** | **N/A** | **N/A** |
| **5** | **21** | **M** | **N/A** | **N/A** | **N/A** | **N/A** | **N/A** | **N/A** | **N/A** | **N/A** | **N/A** | **N/A** | **N/A** | **N/A** |
| **6** | **21** | **M** | **N/A** | **N/A** | **N/A** | **N/A** | **N/A** | **N/A** | **N/A** | **N/A** | **N/A** | **N/A** | **N/A** | **N/A** |
| **7** | **24** | **F** | **26** | **18** | **20** | **19** | **10** | **24** | **12** | **75** | **6** | **20** | **9** | **18** |
| **8** | **27** | **F** | **35** | **17** | **19** | **20** | **10** | **29** | **16** | **71** | **10** | **20** | **8** | **12** |
| **9** | **27** | **F** | **35** | **23** | **20** | **20** | **10** | **16** | **16** | **55** | **9** | **20** | **12** | **13** |
| **10** | **25** | **F** | **35** | **24** | **20** | **19** | **10** | **17** | **12** | **67** | **10** | **20** | **12** | **19** |
| **11** | **25** | **F** | **35** | **24** | **17** | **19** | **9** | **21** | **13** | **69** | **10** | **20** | **12** | **18** |
| **12** | **27** | **F** | **36** | **23** | **19** | **18** | **10** | **19** | **14** | **72** | **10** | **20** | **12** | **16** |
| **13** | **29** | **F** | **28** | **18** | **20** | **19** | **10** | **20** | **11** | **74** | **8** | **20** | **12** | **16** |
| **14** | **28** | **F** | **29** | **22** | **20** | **10** | **10** | **13** | **11** | **47** | **5** | **20** | **11** | **14** |
| **15** | **22** | **F** | **29** | **15** | **18** | **20** | **10** | **18** | **15** | **59** | **7** | **19** | **11** | **14** |
| **16** | **20** | **F** | **22** | **20** | **17** | **19** | **9** | **13** | **16** | **65** | **7** | **20** | **12** | **13** |
| **17** | **22** | **M** | **33** | **19** | **20** | **19** | **10** | **26** | **12** | **69** | **9** | **20** | **12** | **20** |
| **18** | **23** | **M** | **36** | **21** | **20** | **20** | **10** | **25** | **15** | **76** | **10** | **20** | **10** | **18** |
| **19** | **26** | **M** | **29** | **21** | **19** | **18** | **10** | **16** | **12** | **62** | **7** | **20** | **9** | **15** |
| **20** | **22** | **M** | **33** | **N/A** | **19** | **18** | **10** | **19** | **11** | **66** | **10** | **19** | **8** | **17** |
| **21** | **21** | **M** | **35** | **20** | **19** | **20** | **10** | **16** | **14** | **60** | **9** | **20** | **12** | **15** |
| **22** | **20** | **M** | **38** | **23** | **20** | **18** | **10** | **20** | **16** | **71** | **6** | **20** | **12** | **16** |
| **23** | **25** | **M** | **34** | **20** | **20** | **20** | **10** | **22** | **14** | **71** | **8** | **20** | **12** | **20** |
| **24** | **26** | **M** | **33** | **19** | **20** | **17** | **10** | **17** | **11** | **66** | **9** | **20** | **12** | **20** |
| **25** | **27** | **M** | **36** | **21** | **19** | **19** | **9** | **23** | **16** | **79** | **8** | **20** | **11** | **20** |
| **26** | **24** | **M** | **35** | **22** | **20** | **17** | **10** | **24** | **14** | **59** | **10** | **20** | **12** | **18** |
| **27** | **22** | **M** | **34** | **16** | **18** | **20** | **10** | **21** | **15** | **63** | **8** | **19** | **11** | **15** |
| **28** | **31** | **F** | **29** | **23** | **18** | **20** | **8** | **20** | **14** | **57** | **4** | **19** | **10** | **12** |
| **29** | **40** | **F** | **31** | **16** | **20** | **15** | **9** | **16** | **8** | **64** | **9** | **20** | **10** | **20** |
| **30** | **34** | **F** | **36** | **23** | **19** | **19** | **9** | **24** | **13** | **58** | **10** | **20** | **12** | **19** |
| **31** | **35** | **F** | **20** | **19** | **19** | **14** | **10** | **16** | **11** | **68** | **4** | **19** | **11** | **17** |
| **32** | **31** | **F** | **N/A** | **N/A** | **N/A** | **N/A** | **N/A** | **N/A** | **N/A** | **N/A** | **N/A** | **N/A** | **N/A** | **N/A** |
| **33** | **33** | **F** | **32** | **16** | **19** | **19** | **10** | **20** | **10** | **60** | **7** | **20** | **9** | **16** |
| **34** | **33** | **F** | **30** | **17** | **17** | **20** | **10** | **18** | **15** | **56** | **8** | **20** | **7** | **16** |
| **35** | **37** | **F** | **32** | **19** | **19** | **17** | **10** | **17** | **12** | **66** | **10** | **19** | **11** | **16** |
| **36** | **39** | **F** | **33** | **21** | **20** | **16** | **9** | **22** | **11** | **75** | **9** | **20** | **12** | **18** |
| **37** | **31** | **F** | **34** | **20** | **18** | **20** | **10** | **20** | **12** | **71** | **8** | **19** | **12** | **17** |
| **38** | **39** | **M** | **30** | **22** | **13** | **20** | **10** | **19** | **14** | **51** | **7** | **20** | **12** | **13** |
| **39** | **32** | **M** | **33** | **23** | **15** | **20** | **10** | **13** | **16** | **60** | **9** | **20** | **12** | **12** |
| **40** | **38** | **M** | **28** | **14** | **18** | **17** | **10** | **12** | **11** | **60** | **7** | **20** | **10** | **17** |
| **41** | **38** | **M** | **31** | **18** | **20** | **20** | **10** | **21** | **12** | **62** | **4** | **19** | **8** | **15** |
| **42** | **36** | **M** | **32** | **19** | **17** | **18** | **10** | **14** | **11** | **64** | **9** | **20** | **11** | **14** |
| **43** | **32** | **M** | **32** | **20** | **16** | **18** | **10** | **19** | **16** | **66** | **7** | **19** | **11** | **15** |
| **44** | **34** | **M** | **35** | **17** | **20** | **20** | **10** | **13** | **12** | **53** | **6** | **19** | **10** | **14** |
| **45** | **35** | **M** | **29** | **11** | **19** | **20** | **10** | **13** | **14** | **61** | **6** | **20** | **5** | **20** |
| **46** | **35** | **M** | **37** | **24** | **17** | **20** | **10** | **22** | **16** | **66** | **10** | **20** | **12** | **17** |
| **47** | **31** | **M** | **28** | **21** | **18** | **20** | **10** | **21** | **13** | **60** | **7** | **20** | **11** | **9** |
| **48** | **45** | **M** | **28** | **14** | **20** | **20** | **10** | **22** | **16** | **54** | **1** | **20** | **5** | **19** |
| **49** | **45** | **M** | **29** | **21** | **19** | **20** | **10** | **22** | **10** | **65** | **8** | **20** | **12** | **19** |
| **50** | **43** | **M** | **28** | **21** | **20** | **18** | **10** | **31** | **15** | **35** | **5** | **19** | **11** | **17** |
| **51** | **45** | **M** | **28** | **18** | **20** | **20** | **10** | **17** | **15** | **64** | **5** | **19** | **9** | **16** |
| **52** | **44** | **M** | **35** | **22** | **20** | **17** | **10** | **22** | **13** | **69** | **7** | **19** | **12** | **18** |
| **53** | **40** | **M** | **30** | **18** | **18** | **20** | **9** | **26** | **9** | **57** | **6** | **19** | **11** | **15** |
| **54** | **49** | **F** | **20** | **14** | **17** | **17** | **10** | **26** | **13** | **53** | **3** | **19** | **7** | **12** |
| **55** | **46** | **F** | **33** | **21** | **20** | **15** | **10** | **21** | **11** | **61** | **6** | **20** | **10** | **15** |
| **56** | **43** | **F** | **30** | **11** | **20** | **15** | **9** | **19** | **11** | **57** | **0** | **20** | **5** | **12** |
| **57** | **47** | **F** | **31** | **23** | **18** | **20** | **9** | **19** | **14** | **61** | **8** | **19** | **11** | **7** |
| **58** | **46** | **F** | **30** | **19** | **19** | **20** | **9** | **16** | **14** | **59** | **7** | **20** | **12** | **10** |
| **59** | **47** | **F** | **28** | **14** | **20** | **17** | **9** | **17** | **9** | **48** | **6** | **20** | **10** | **15** |
| **60** | **49** | **F** | **22** | **20** | **19** | **14** | **10** | **21** | **9** | **60** | **4** | **18** | **10** | **11** |
| **61** | **45** | **F** | **28** | **22** | **20** | **19** | **10** | **20** | **10** | **62** | **6** | **20** | **11** | **17** |
| **62** | **49** | **F** | **28** | **17** | **20** | **18** | **10** | **16** | **8** | **43** | **9** | **20** | **10** | **15** |
| **63** | **49** | **F** | **31** | **21** | **20** | **20** | **10** | **10** | **14** | **53** | **9** | **20** | **12** | **14** |
| **64** | **49** | **M** | **21** | **12** | **16** | **20** | **10** | **23** | **10** | **53** | **4** | **17** | **11** | **16** |
| **65** | **46** | **M** | **23** | **19** | **16** | **19** | **10** | **14** | **8** | **49** | **5** | **19** | **10** | **15** |
| **66** | **40** | **M** | **25** | **23** | **20** | **19** | **10** | **20** | **16** | **78** | **5** | **19** | **12** | **20** |
| **67** | **44** | **M** | **29** | **18** | **16** | **19** | **8** | **18** | **8** | **59** | **7** | **20** | **12** | **14** |
| **68** | **43** | **M** | **27** | **22** | **16** | **20** | **10** | **14** | **11** | **58** | **6** | **20** | **11** | **13** |
| **69** | **49** | **M** | **30** | **13** | **20** | **18** | **10** | **18** | **11** | **56** | **8** | **20** | **8** | **17** |
| **70** | **42** | **M** | **27** | **20** | **19** | **17** | **10** | **22** | **16** | **63** | **9** | **20** | **11** | **14** |
| **71** | **47** | **M** | **26** | **15** | **14** | **19** | **10** | **17** | **11** | **44** | **4** | **20** | **11** | **7** |
| **72** | **40** | **M** | **33** | **18** | **20** | **20** | **9** | **14** | **11** | **75** | **10** | **20** | **9** | **19** |
| **73** | **42** | **M** | **30** | **20** | **19** | **20** | **10** | **22** | **9** | **60** | **6** | **20** | **11** | **18** |
| **74** | **52** | **F** | **30** | **21** | **16** | **19** | **9** | **15** | **12** | **58** | **8** | **20** | **9** | **7** |
| **75** | **52** | **F** | **29** | **22** | **18** | **20** | **10** | **23** | **10** | **66** | **7** | **17** | **12** | **10** |
| **76** | **56** | **F** | **27** | **10** | **17** | **16** | **9** | **18** | **8** | **52** | **5** | **20** | **4** | **13** |
| **77** | **56** | **F** | **29** | **21** | **17** | **20** | **9** | **21** | **16** | **66** | **6** | **19** | **12** | **16** |
| **78** | **57** | **F** | **25** | **16** | **15** | **18** | **9** | **16** | **10** | **50** | **2** | **20** | **9** | **5** |
| **79** | **52** | **F** | **26** | **15** | **18** | **18** | **9** | **14** | **16** | **61** | **6** | **18** | **9** | **13** |
| **80** | **57** | **F** | **28** | **15** | **18** | **19** | **10** | **22** | **12** | **63** | **6** | **20** | **12** | **16** |
| **81** | **54** | **F** | **33** | **21** | **13** | **15** | **10** | **16** | **8** | **60** | **8** | **20** | **11** | **12** |
| **82** | **57** | **F** | **29** | **21** | **19** | **18** | **10** | **20** | **10** | **44** | **7** | **18** | **12** | **17** |
| **83** | **56** | **F** | **24** | **19** | **17** | **19** | **10** | **25** | **12** | **61** | **6** | **19** | **12** | **9** |
| **84** | **52** | **M** | **34** | **22** | **19** | **20** | **10** | **23** | **12** | **66** | **8** | **19** | **12** | **15** |
| **85** | **50** | **M** | **32** | **18** | **20** | **20** | **10** | **12** | **9** | **60** | **10** | **20** | **11** | **17** |
| **86** | **55** | **M** | **33** | **23** | **20** | **20** | **10** | **17** | **16** | **78** | **10** | **20** | **12** | **15** |
| **87** | **53** | **M** | **29** | **16** | **19** | **19** | **10** | **19** | **12** | **45** | **8** | **19** | **7** | **13** |
| **88** | **55** | **M** | **23** | **16** | **19** | **16** | **8** | **14** | **15** | **49** | **4** | **17** | **8** | **12** |
| **89** | **51** | **M** | **26** | **15** | **13** | **20** | **10** | **10** | **14** | **62** | **2** | **16** | **7** | **9** |
| **90** | **50** | **M** | **27** | **15** | **14** | **20** | **10** | **15** | **11** | **56** | **7** | **20** | **9** | **6** |
| **91** | **56** | **M** | **32** | **19** | **15** | **18** | **16** | **13** | **16** | **65** | **N/A** | **20** | **12** | **12** |
| **92** | **57** | **M** | **32** | **18** | **11** | **19** | **10** | **13** | **16** | **60** | **9** | **20** | **11** | **8** |
| **93** | **54** | **M** | **34** | **21** | **15** | **20** | **9** | **19** | **11** | **64** | **9** | **20** | **12** | **7** |
| **94** | **65** | **F** | **28** | **12** | **18** | **14** | **10** | **24** | **16** | **53** | **6** | **19** | **5** | **5** |
| **95** | **60** | **F** | **30** | **17** | **18** | **15** | **10** | **22** | **16** | **55** | **8** | **17** | **11** | **4** |
| **96** | **62** | **F** | **26** | **14** | **12** | **15** | **8** | **19** | **12** | **59** | **6** | **17** | **7** | **4** |
| **97** | **60** | **F** | **30** | **21** | **17** | **19** | **7** | **19** | **12** | **52** | **6** | **20** | **11** | **16** |
| **98** | **64** | **F** | **25** | **20** | **11** | **10** | **10** | **15** | **11** | **61** | **7** | **19** | **12** | **11** |
| **99** | **64** | **F** | **27** | **16** | **16** | **18** | **10** | **16** | **11** | **51** | **4** | **20** | **10** | **14** |
| **100** | **63** | **F** | **21** | **21** | **14** | **19** | **10** | **10** | **8** | **51** | **5** | **18** | **11** | **16** |
| **101** | **61** | **F** | **27** | **16** | **16** | **19** | **10** | **20** | **13** | **64** | **5** | **19** | **3** | **6** |
| **102** | **63** | **F** | **27** | **22** | **16** | **17** | **10** | **14** | **10** | **45** | **5** | **19** | **11** | **8** |
| **103** | **61** | **F** | **26** | **12** | **13** | **16** | **10** | **24** | **8** | **56** | **5** | **18** | **9** | **6** |
| **104** | **60** | **M** | **26** | **22** | **13** | **12** | **10** | **17** | **9** | **53** | **6** | **20** | **9** | **6** |
| **105** | **64** | **M** | **21** | **21** | **14** | **19** | **10** | **10** | **8** | **51** | **5** | **18** | **11** | **16** |
| **106** | **60** | **M** | **26** | **17** | **15** | **18** | **10** | **25** | **11** | **82** | **5** | **20** | **11** | **11** |
| **107** | **62** | **M** | **25** | **18** | **10** | **16** | **10** | **19** | **8** | **50** | **5** | **19** | **11** | **5** |
| **108** | **64** | **M** | **27** | **14** | **13** | **15** | **10** | **20** | **12** | **51** | **8** | **20** | **10** | **10** |
| **109** | **64** | **M** | **27** | **15** | **13** | **16** | **10** | **14** | **9** | **57** | **6** | **18** | **11** | **8** |
| **110** | **61** | **M** | **29** | **20** | **14** | **20** | **10** | **21** | **12** | **61** | **8** | **19** | **11** | **15** |
| **111** | **65** | **M** | **25** | **17** | **15** | **14** | **10** | **19** | **9** | **50** | **7** | **20** | **11** | **13** |
| **112** | **67** | **M** | **21** | **10** | **12** | **15** | **9** | **8** | **10** | **36** | **3** | **18** | **4** | **11** |
| **113** | **63** | **M** | **21** | **16** | **16** | **19** | **10** | **11** | **11** | **59** | **5** | **16** | **11** | **10** |
| **114** | **56** | **M** | **N/A** | **N/A** | **N/A** | **N/A** | **N/A** | **N/A** | **N/A** | **N/A** | **N/A** | **N/A** | **N/A** | **N/A** |
| **115** | **64** | **M** | **N/A** | **N/A** | **N/A** | **N/A** | **N/A** | **N/A** | **N/A** | **N/A** | **N/A** | **N/A** | **N/A** | **N/A** |
| **116** | **76** | **M** | **N/A** | **N/A** | **N/A** | **N/A** | **N/A** | **N/A** | **N/A** | **N/A** | **N/A** | **N/A** | **N/A** | **N/A** |
| **117** | **76** | **M** | **N/A** | **N/A** | **N/A** | **N/A** | **N/A** | **N/A** | **N/A** | **N/A** | **N/A** | **N/A** | **N/A** | **N/A** |
| **118** | **73** | **M** | **N/A** | **N/A** | **N/A** | **N/A** | **N/A** | **N/A** | **N/A** | **N/A** | **N/A** | **N/A** | **N/A** | **N/A** |
| **119** | **55** | **M** | **N/A** | **N/A** | **N/A** | **N/A** | **N/A** | **N/A** | **N/A** | **N/A** | **N/A** | **N/A** | **N/A** | **N/A** |
| **120** | **72** | **M** | **N/A** | **N/A** | **N/A** | **N/A** | **N/A** | **N/A** | **N/A** | **N/A** | **N/A** | **N/A** | **N/A** | **N/A** |
| **121** | **59** | **M** | **N/A** | **N/A** | **N/A** | **N/A** | **N/A** | **N/A** | **N/A** | **N/A** | **N/A** | **N/A** | **N/A** | **N/A** |
| **122** | **73** | **M** | **N/A** | **N/A** | **N/A** | **N/A** | **N/A** | **N/A** | **N/A** | **N/A** | **N/A** | **N/A** | **N/A** | **N/A** |
| **123** | **57** | **M** | **N/A** | **N/A** | **N/A** | **N/A** | **N/A** | **N/A** | **N/A** | **N/A** | **N/A** | **N/A** | **N/A** | **N/A** |
| **124** | **71** | **M** | **N/A** | **N/A** | **N/A** | **N/A** | **N/A** | **N/A** | **N/A** | **N/A** | **N/A** | **N/A** | **N/A** | **N/A** |
| **125** | **65** | **M** | **N/A** | **N/A** | **N/A** | **N/A** | **N/A** | **N/A** | **N/A** | **N/A** | **N/A** | **N/A** | **N/A** | **N/A** |
| **126** | **74** | **F** | **N/A** | **N/A** | **N/A** | **N/A** | **N/A** | **N/A** | **N/A** | **N/A** | **N/A** | **N/A** | **N/A** | **N/A** |
| **127** | **76** | **M** | **N/A** | **N/A** | **N/A** | **N/A** | **N/A** | **N/A** | **N/A** | **N/A** | **N/A** | **N/A** | **N/A** | **N/A** |
| **128** | **71** | **M** | **N/A** | **N/A** | **N/A** | **N/A** | **N/A** | **N/A** | **N/A** | **N/A** | **N/A** | **N/A** | **N/A** | **N/A** |
| **129** | **73** | **M** | **N/A** | **N/A** | **N/A** | **N/A** | **N/A** | **N/A** | **N/A** | **N/A** | **N/A** | **N/A** | **N/A** | **N/A** |
| **130** | **71** | **M** | **N/A** | **N/A** | **N/A** | **N/A** | **N/A** | **N/A** | **N/A** | **N/A** | **N/A** | **N/A** | **N/A** | **N/A** |
| **131** | **76** | **M** | **N/A** | **N/A** | **N/A** | **N/A** | **N/A** | **N/A** | **N/A** | **N/A** | **N/A** | **N/A** | **N/A** | **N/A** |
| **132** | **71** | **F** | **N/A** | **N/A** | **N/A** | **N/A** | **N/A** | **N/A** | **N/A** | **N/A** | **N/A** | **N/A** | **N/A** | **N/A** |
| **133** | **70** | **M** | **N/A** | **N/A** | **N/A** | **N/A** | **N/A** | **N/A** | **N/A** | **N/A** | **N/A** | **N/A** | **N/A** | **N/A** |
| **134** | **74** | **M** | **N/A** | **N/A** | **N/A** | **N/A** | **N/A** | **N/A** | **N/A** | **N/A** | **N/A** | **N/A** | **N/A** | **N/A** |
| **135** | **72** | **F** | **N/A** | **N/A** | **N/A** | **N/A** | **N/A** | **N/A** | **N/A** | **N/A** | **N/A** | **N/A** | **N/A** | **N/A** |
| **136** | **70** | **M** | **N/A** | **N/A** | **N/A** | **N/A** | **N/A** | **N/A** | **N/A** | **N/A** | **N/A** | **N/A** | **N/A** | **N/A** |
| **137** | **76** | **M** | **N/A** | **N/A** | **N/A** | **N/A** | **N/A** | **N/A** | **N/A** | **N/A** | **N/A** | **N/A** | **N/A** | **N/A** |
| **138** | **77** | **M** | **N/A** | **N/A** | **N/A** | **N/A** | **N/A** | **N/A** | **N/A** | **N/A** | **N/A** | **N/A** | **N/A** | **N/A** |
| **139** | **76** | **M** | **N/A** | **N/A** | **N/A** | **N/A** | **N/A** | **N/A** | **N/A** | **N/A** | **N/A** | **N/A** | **N/A** | **N/A** |
| **140** | **79** | **M** | **N/A** | **N/A** | **N/A** | **N/A** | **N/A** | **N/A** | **N/A** | **N/A** | **N/A** | **N/A** | **N/A** | **N/A** |
| **141** | **76** | **F** | **N/A** | **N/A** | **N/A** | **N/A** | **N/A** | **N/A** | **N/A** | **N/A** | **N/A** | **N/A** | **N/A** | **N/A** |
| **142** | **79** | **F** | **N/A** | **N/A** | **N/A** | **N/A** | **N/A** | **N/A** | **N/A** | **N/A** | **N/A** | **N/A** | **N/A** | **N/A** |
| **143** | **70** | **F** | **N/A** | **N/A** | **N/A** | **N/A** | **N/A** | **N/A** | **N/A** | **N/A** | **N/A** | **N/A** | **N/A** | **N/A** |

M: Male, F: Female, N/A: Not Available

**Table S2. Number of participants by age group, sex, and scanner (numbers of female participants are shown in parentheses)**

| Age group | Total | Scanner | | | | | |
| --- | --- | --- | --- | --- | --- | --- | --- |
|  |  | YCU  Aquiduo | YCU  Celesteion | Keio  Biograph mCT | Fukui  Signa  PET/MRI | Kyushu  Biograph mCT | Kyushu  Biograph Vision |
| 20s | 27(10) | 6(0) | 5(2) | 0 | 10(4) | 4(3) | 2(1) |
| 30s | 20(10) | 9(4) | 7(4) | 0 | 0 | 1(1) | 3(1) |
| 40s | 26(10) | 9(2) | 15(7) | 0 | 0 | 2(1) | 0 |
| 50s | 24(10) | 1(0) | 16(10) | 3(0) | 0 | 1(0) | 3(0) |
| 60s | 22(10) | 0 | 19(8) | 1(0) | 1(1) | 1(1) | 0 |
| 70s | 24(6) | 0 | 19(6) | 5(0) | 0 | 0 | 0 |

YCU: Yokohama City University
